# Risk of recurrent cardiovascular events in coronary artery disease patients with Type D personality

**DOI:** 10.3389/fpsyg.2023.1119146

**Published:** 2023-03-28

**Authors:** Kristin Stensland Torgersen, Elise Christine Bjørkholen Sverre, Harald Weedon-Fekjær, Ole A. Andreassen, John Munkhaugen, Toril Dammen

**Affiliations:** ^1^Department of Behavioural Medicine, Faculty of Medicine, University of Oslo, Oslo, Norway; ^2^Institute of Clinical Medicine, University of Oslo, Oslo, Norway; ^3^Department of Medicine, Drammen Hospital, Drammen, Norway; ^4^Oslo Center for Biostatistics and Epidemiology, Research Support Services, Oslo University Hospital, Oslo, Norway; ^5^NORMENT: Norwegian Centre for Mental Disorders Research, University of Oslo and Oslo University Hospital, Oslo, Norway; ^6^Section of Psychiatric Treatment Research, Division of Mental Health and Addiction, Oslo University Hospital, Oslo, Norway

**Keywords:** coronary artery disease, secondary prevention, cardiovascular risk factors (CVRFs), recurrent cardiovascular events, Type D personality, anxiety, depression

## Abstract

**Introduction:**

Data on the association between Type D personality, its traits negative affectivity (NA) and social inhibition (SI), and risk of major adverse cardiac events (MACE) in coronary outpatients is sparse. Furthermore, the associations between Type D subgroups and cardiovascular risk factors are largely unknown.

**Methods:**

We investigated i) Type D personality, NA and SI and risk of recurrent MACE, and ii) the relationship between Type D subgroups and risk factors in a coronary population. This prospective cohort study included 1083 patients` median 16 months after a myocardial infarction and/or a revascularization procedure who were followed-up for 4.2 (SD 0.4) years. Type D personality was assessed by DS14. Anxiety and depression, statin adherence, and risk factors were assessed by patients’ self-report and a clinical examination with blood samples. MACE, defined as cardiovascular death, myocardial infarction, revascularization, stroke or heart failure, were obtained from hospital records from index event to end of study lasting 5.7 years. Data were analyzed by Cox proportional hazard regression.

**Results:**

In all, 352 MACE occurred in 230 patients after average 4.2 years follow-up. Higher NA score was associated with MACE after adjustment for age, risk factors and comorbidity (HR 1.02 per unit increase, 95% CI 1.00-1.05), whereas we found a weaker, not statistically significant estimated effect of higher SI score. After additional adjustment for symptoms of anxiety and depression, we found a weaker, not statistically significant association between NA and MACE (HR 1.01 per unit increase, 95% CI 0.98-1.05). Low statin adherence and smoking were more prevalent in the Type D and high NA group.

**Discussion:**

Our results indicate that the NA trait is related to worse prognosis in outpatients with coronary artery disease.

## Introduction

Patients with Type D (distressed) personality are characterized by simultaneously having high levels of negative affectivity (NA)–the tendency to experience negative emotions, as well as high levels of social inhibition (SI)—the tendency to inhibit self-expression in social interactions (Denollet et al., 2000). Type D personality is assessed by the self-report questionnaire DS14 (Denollet, 2005). Type D personality is prevalent in coronary artery disease (CAD) patients, ranging from 13% to 50% (Mommersteeg et al., 2010; Christodoulou et al., 2013; Kupper and Denollet, 2018). Although the most recent European guidelines on Cardiovascular Disease (CVD) prevention recognize Type D personality and other psychosocial factors as risk modifiers (Visseren et al., 2021), the association between Type D personality and risk for recurrent CVD events remains controversial (Grande et al., 2012).

Earlier studies reported poorer prognosis in terms of recurrent CVD events and mortality among CAD patients with Type D personality compared to those without Type D (Denollet et al., 1995, 1996, 2000, 2006a,b; Denollet and Brutsaert, 1998; Pedersen et al., 2004, 2007; Denollet and Pedersen, 2008; Martens et al., 2010; Du et al., 2016; Imbalzano et al., 2018; Wang et al., 2018), whereas more recent studies find conflicting results (Grande et al., 2011; Meyer et al., 2014; Conden et al., 2017; Raykh et al., 2021). Most previous studies have been conducted at the time of hospitalization for an acute coronary event or a revascularization procedure (Denollet et al., 1995; Denollet and Brutsaert, 1998; Denollet et al., 2000; Grande et al., 2011; Meyer et al., 2014; Vukovic et al., 2014; Garcia-Retamero et al., 2016; Conden et al., 2017; Imbalzano et al., 2018) or in cardiac rehabilitation units (Denollet et al., 2006a; Grande et al., 2011) shortly after hospital discharge. Knowledge about the association between Type D personality and prognosis in CAD outpatients’ longer time after the event is limited. Because Type D assessment may be affected by the psychological reaction with potentially higher levels of distress in the acute state compared to that of the chronic state, there is a need to study the association between Type D personality and CVD prognosis also in CAD outpatients` longer time after hospitalization. A general practitioner mainly follow up these patients, and if those who score positive for Type D personality have poor prognosis, screening may be considered. It is still unclear which aspects of Type D personality that may relates to prognosis; whether it is Type D personality *per se*, or NA or SI. There are some indications that NA and SI may have independent and different contributions to CVD prognosis in CAD patients. Few studies have explored the associations between both Type D, and NA and SI, and risk of CVD events (Denollet et al., 2013a; Wang et al., 2018). Of these, some have only identified an association between elevated NA scores and poor outcome NA (Wang et al., 2018) whereas others have identified better cardiovascular outcomes in those with high SI and no prognostic impact on prognosis of Type D or NA (Meyer et al., 2014; Dulfer et al., 2015). Hence, there is a need to study the relative importance of these Type D variables and their prognostic potential in CAD patients.

Depression is also a known prevalent risk factor for CVD morbidity and mortality in patients with established CAD (Carney and Freedland, 2017), often co-occurring with Type D personality (Al-Qezweny et al., 2016). It has been discussed whether Type D personality is associated with poor prognosis primarily through depression, and if these are separate or overlapping constructs (Marchesi et al., 2014). It is therefore important to adjust for depression to elucidate the independent prognostic contribution of Type D personality. Studies controlling for symptoms of depression have shown conflicting results. Whereas most studies have reported an independent significant associations between Type D personality, depression, and poor prognosis in CAD patients (Denollet et al., 1995, 1996, 2000, 2006b; Denollet and Brutsaert, 1998; Denollet and Pedersen, 2008; Martens et al., 2010; Vukovic et al., 2014; Wang et al., 2018), others have failed to find such an association (Grande et al., 2011). Symptoms of anxiety are associated with both Type D personality (Kupper and Denollet, 2014), depression as well as with poor cardiovascular prognosis in CAD patients (Al-Qezweny et al., 2016). To the best of our knowledge, only two prior studies with Type D assessments up to 6 months after the cardiac event have adjusted for symptoms of anxiety (Denollet and Brutsaert, 1998; Denollet et al., 2006a). Both studies reported an independent effect of Type D on cardiac prognosis (Denollet and Brutsaert, 1998; Denollet et al., 2006a). It remains to be explored if Type D personality influences prognosis in CAD outpatients` longer time after the acute event, after controlling for both anxiety and depression.

Different biological and behavioral pathways have been proposed to explain the adverse prognosis in CAD patients with Type D personality including unfavorable lifestyle, poor adherence to medication, low participation rates in cardiac rehabilitation or effects on the immune system (C-reactive protein; Denollet and Conraads, 2011). Few previous follow-up studies in CAD patients have reported associations between these factors and Type D personality (Pedersen et al., 2004; Majaluoma et al., 2020), and the association in subgroups according to high NA/low SI and high SI/low NA has yet to be investigated. This knowledge will improve our understanding of the behavioral links between Type D personality and cardiac prognosis. It may also explain why SI has been associated both with better (Meyer et al., 2014) and worse cardiovascular outcome in some studies (Conden et al., 2017), whereas NA has been the major contributor in other studies (Denollet and Brutsaert, 1998; Wang et al., 2018).

This study aimed to investigate (i) the association between Type D personality and the Type D traits of NA and SI and risk of recurrent MACE after adjusting for anxiety and depression, and (ii) the relationship between Type D subgroups and CVD risk factors, depression and anxiety in a coronary outpatient population.

## Materials and methods

### Design and population

**Figure 1:**
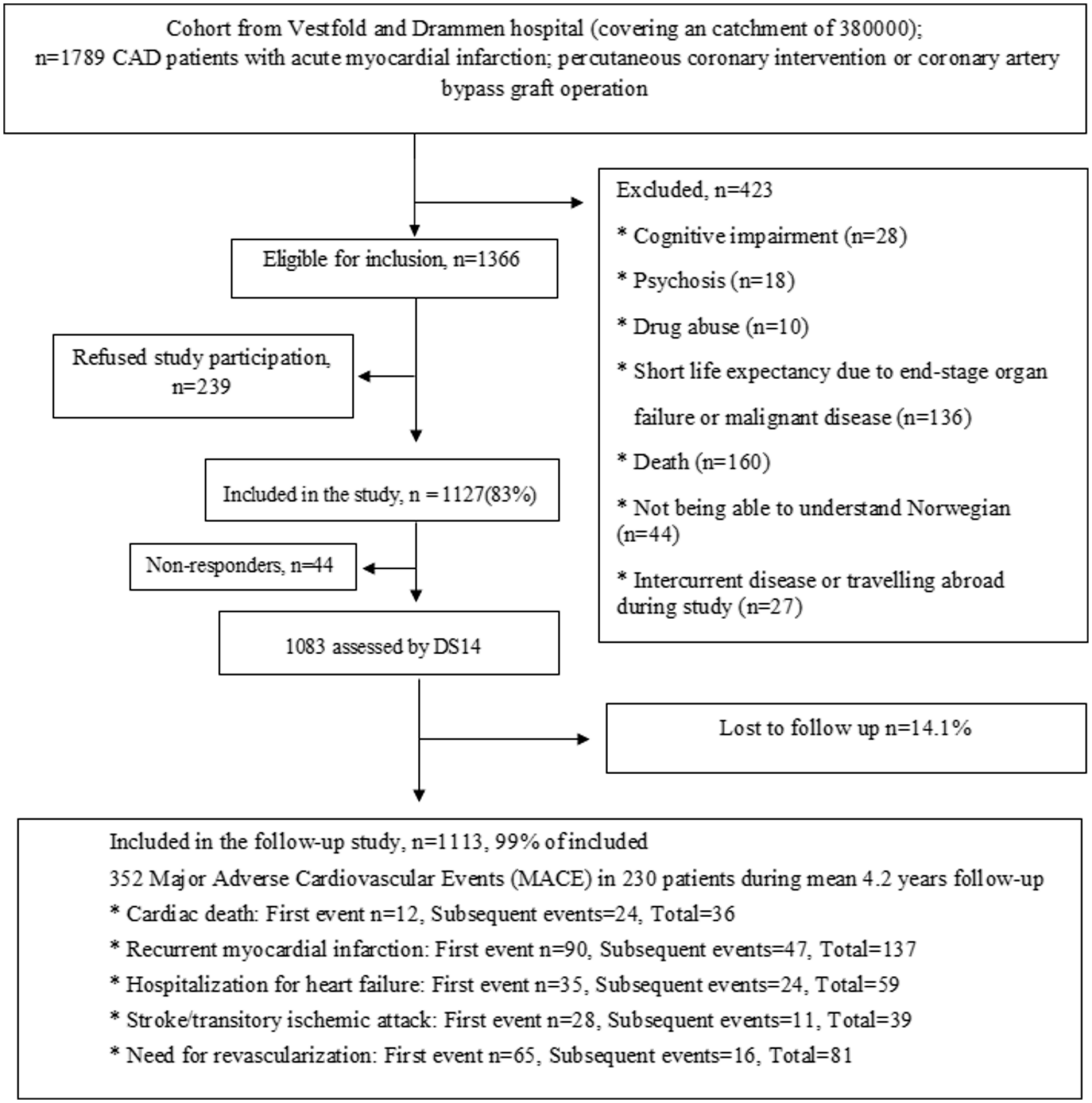
Study flow chart.

In this pre-planned study, data were obtained from the NORwegian CORonary (NOR-COR) prevention study (ClinicalTrials.gov: ID NCT02309255) conducted at two Norwegian hospitals (Drammen and Vestfold). The design, methods and baseline characteristics of the NOR-COR study have been described in detail elsewhere (Munkhaugen et al., 2016). The study flow chart including inclusion and exclusion criteria is shown in Figure 1. In brief, 1,789 consecutive patients aged 18–80 years with an index coronary event (myocardial infarction (MI) and/or coronary revascularization) in 2011–2014 were identified from hospital discharge lists. Of these, 423 were excluded, resulting in 1,366 eligible patients. With a participation rate of 83%, 1,127 patients were included for baseline assessments during 2014–2015 median 16 months (range 2–36) after the coronary index event. The index event was defined as the last coronary event prior to inclusion.

At baseline, the participants attended a clinical examination with blood sample collection and completed a comprehensive self-report questionnaire. In all, 1,083 patients completed the DS14 (Denollet, 2005) for Type D personality (Munkhaugen et al., 2016). Follow-up data on recurrent CVD events were collected from medical records after a mean follow-up of 4.2 years (standard deviation (SD) 0.4) between October and November 2018. Complete follow-up data were missing in only 14 (1%) of the patients, resulting in 1,069 patients.

The catchment area of two participating hospitals covers an area with a population of 380,000 inhabitants corresponding to 7.4% of the Norwegian population with a representative blend of city and rural districts. The population is mostly representative of Norwegians with respect to education, economy, age distribution, morbidity, and mortality (Munkhaugen et al., 2016).

### Variables

#### Major adverse cardiovascular events

Two experienced cardiac researchers collected data on the pre-defined composite primary outcome Major adverse cardiovascular events (MACE) from the patients’ hospital records from 10th October to 30th November 2018. MACE comprised CVD death, readmission for MI, a new revascularization procedure due to angina, or hospital admission for stroke/transitory ischemic attacks or heart failure.

#### Type D personality assessment

Type D personality was assessed by DS14 (Denollet, 2005), a 14-item measure, each item is answered on a 5-point Likert scale from 0 to 4. The scale consists of two 7-item subscales assessing NA and SI. Type D personality is defined as having a score ≥ 10 on both the NA and SI subscales (Lloyd-Jones et al., 2010). Furthermore, we categorized the patients into four subgroups: NA < 10/SI < 10 (non-Type D), NA ≥ 10/SI < 10 (high NA), NA < 10/SI ≥ 10 (high SI) and NA ≥ 10/SI ≥ 10 (Type D). The Norwegian version of the DS14 has been validated in a sample of CAD patients, with acceptable psychometric properties with Cronbach α of 0.87 for NA and 0.83 for SI (Bergvik et al., 2010) and with a 4-week test–retest reliability of 0.74–1.0 (Peersen et al., 2017).

#### Clinical and psychological variables

The following clinical variables were obtained from medical records at baseline: Age, sex, coronary history and treatment, diabetes, and CVD comorbidity, and participation in the cardiac rehabilitation program. The comprehensive self-report questionnaire with highly acceptable reproducibility for all key items and instruments (Peersen et al., 2017) included level of education (low < 12 years), living alone (yes/no), smoking history (current), low physical activity (<30 min moderate activity 3 times a week), adherence to statins last week (Munkhaugen et al., 2016). It also covered symptoms of depression and anxiety assessed by the Hospital Anxiety and Depression Rating Scale (HADS; Zigmond and Snaith, 1983), a 14-item self-report questionnaire, consisting of 2 seven-item subscales that assess anxiety (HADS-A) and depressive symptoms (HADS-D). The scale has demonstrated good psychometric properties in CAD patients (Bjelland et al., 2002). The Norwegian version of HADS has been reputed with good internal consistency and acceptable validity across studies (Leiknes and Siqveland, 2016). The 4-week test–retest reliability was 0.92 for HADS-A and 0.94 for HADS-D in the baseline study (Peersen et al., 2017).

Low-density lipoprotein (LDL) cholesterol and C-reactive protein (CRP) from non-fasting venous blood samples were analyzed on an Architect ci16200 (Abbott Laboratories, United States) at Drammen hospital to avoid inter-laboratory bias. Systolic blood pressure was measured with standardized procedure using a validated digital sphygmomanometer (Welch Allyn Connex ProBP 3400) and waist circumference with a non-stretchable tape (Seca 201, Seca, Birmingham, United Kingdom) at the clinical examination.

### Statistical analyses

Descriptive statistics were presented as frequencies and percentages for proportions, and mean with standard deviations (SD) for continuous variables. Differences between groups were given by 95% confidence intervals based on the t-distribution, and tested by χ^2^ tests for categorical variables and *t*-tests for continuous variables. The one-way analysis of variance (ANOVA) was used to determine whether there were any statistically significant differences between the means of three or more groups and likelihood ratio test for multi-nominal logistic regression was used for proportions. Internal consistency of scales was assessed by Cronbach’s alpha. Hazard ratios (HR) with 95% confidence interval (CI) were calculated by Cox proportional hazards regression for the first MACE after study inclusion. Analysis time in the Cox model was set from the time of the index coronary event, to adjust for baseline variations in risk by time since the index coronary event. Patients were followed until the date of death or the end of study (1st December 2018). In addition to the main analysis, data were also analyzed using all MACE events to evaluate whether results were consistent with the increased number of end-points and a more biologically mixed dataset.

We defined four hierarchic models adjusting for different sets of co-variables. In model 1, we adjusted for age. In model 2, we adjusted for risk factors given as smoking, LDL cholesterol, physical activity, and systolic blood pressure, in addition to age. In model 3, we added adjustment for somatic comorbidity given as stroke, peripheral artery disease and kidney failure. In model 4, we also adjusted for symptoms of anxiety and depression. Only variables with an univariate value of p less than 0.1 were included in the models. All Cox regression analyses were stratified for prior CAD before the index event, as patients with established CAD prior to the index event were assumed to have another risk profile by study time.

Most applied variables had few missing values (range: 0%–10%). However, in the multivariable Cox regression analysis the combination of missing values for different covariates resulted in 262 excluded patients (including 50 patients with a MACE). These missing cases lowered the statistical power and could potentially have introduced a systematic bias. Hence, we performed iterative Markov chain Monte Carlo multiple imputation under a missing at random assumption (Donders et al., 2006). For statistical analyses Stata version 15 (StataCorp LLC, College Station, United States) was used.

## Results

The sample consisted of 79% men and mean age was 61.5 (SD 9.6) years. MI was an index event for 78, and 22% had angina with angiography-verified stenosis. Type D personality was found in 18% (*n* = 197). Patients that did not respond to the DS14 questionnaire reported higher mean HADS-A scores than the other participants, 6.7 (SD 4.6) vs. 4.7 (SD 3.7), value of *p* = 0.01. Otherwise, there were no marked differences between these groups (Supplementary Table 1).

Differences in characteristics between patients with and without Type D personality at baseline are shown in Table 1. Patients with Type D were significantly younger, more often female, were more likely to smoke, and had lower systolic blood pressure and higher scores on symptoms of depression and anxiety.

**Table 1:** Baseline characteristics of Type-D and non-Type D patients.

	All patients (*n* = 1,083)	Type D (*n* = 197)	Non-Type D (*n* = 886)	value of p for difference
**Socio-demographic factors**				
Age at index event, mean (SD)	61.5 (9.6)	59.3 (10.5)	62.1 (9.3)	0.001
Female sex, % (*n*)	21.0 (227)	26.4 (52)	19.8 (175)	0.042
Living alone, % (*n*)	19.2 (194)	22.3 (40)	18.6 (154)	0.251
Low education,^1^ % (*n*)	70.1 (752)	75.6 (146)	68.9 (606)	0.068
**Clinical factors**				
*Coronary index diagnosis*				
Myocardial infarction, % (*n*)	23.8 (258)	25.9 (51)	23.4 (207)	0.880 0.064
Stable or unstable angina, % (*n*)	20.6 (223)	16.2 (32)	21.6 (191)	
Cardiovascular comorbidity	14.4 (156)	16.8 (33)	13.9 (123)	0.313
More than 1 previous coronary event, % (*n*)	29.6 (321)	29.9 (59)	29.6 (262)	0.916
Heart failure, % (*n*)	12.9 (140)	10.2 (20)	13.5 (120)	0.240
Peripheral artery disease, % (*n*)	8.6 (93)	12.7 (25)	7.7 (68)	0.025
Stroke or transient ischemic attack, % (*n*)	6.9 (75)	6.1 (12)	7.1 (63)	0.647
Chronic kidney failure (eGFR < 60 mL/min/1.73 m^2^), % (*n*)	13.3 (132)	15.0 (27)	12.9 (105)	0.467
Participation in cardiac rehabilitation, % (*n*)	53.0 (574)	49.7 (98)	53.7 (476)	0.344
Not taking statins last week, % (*n*)	5.0 (53)	7.3 (14)	4.5 (39)	0.098
Not using statins at inclusion, % (*n*)	8.9 (95)	11.5 (22)	8.3 (73)	0.206
Current smoking,^2^ % (*n*)	20.8 (217)	29.0 (56)	18.9 (161)	0.002
Low physical activity,^3^ % (*n*)	59.4 (639)	64.5 (127)	58.3 (512)	0.127
Systolic blood pressure (mmHg), mean (SD)	138 (19.0)	136 (17.9)	139 (19.2)	0.033
LDL-Cholesterol (mmol/L), mean (SD)	2.1 (0.8)	2.1 (0.8)	2.1 (0.8)	0.977
Diabetes, % (*n*)	16.6 (180)	18.8 (37)	16.1 (143)	0.368
C-reactive protein, mean (SD)	2.5 (2.9)	2.5 (2.9)	2.5 (2.8)	0.937
Central obesity,^4^ % (*n*)	59.4 (579)	63.0 (109)	58.6 (470)	0.306
**Psychological factors**				
DS14 negative affection (score 0–28), mean (SD)	7.0 (5.9)	15.3 (4.1)	5.2 (4.4)	<0.001
DS14 social inhibition (score 0–28), mean (SD)	7.5 (5.7)	14.8 (3.7)	5.9 (4.7)	<0.001
Hospital Anxiety and Depression Score—depression, mean (SD)	3.9 (3.2)	7.0 (3.5)	3.1 (2.6)	<0.001
Hospital Anxiety and Depression Score—anxiety, mean (SD)	4.8 (3.7)	8.4 (3.7)	3.9 (3.1)	<0.001

SD, Standard deviation; *n,* number; LDL, low density lipoprotein; eGFR, estimated glomerular filtration rate.

1 Completion of primary or secondary school only.

2 Smoking at inclusion.

3 Physical activity less than 30 min of moderate activity 2–3 times weekly.

4 Waist circumference ≥ 102 cm in males and ≥ 88 cm in females.

In total, 352 MACE occurred in 230 patients during the mean follow-up period of 4.2 (SD 0.4) years. For Type D personality, we found a non-significant HR of 1.25 for MACE in age-adjusted analyzes (95% CI 0.91–1.71). We found no considerable significant association between MACE and SI scores in either crude or adjusted analyses (Table 2). Higher NA scores were associated with MACE in age adjusted analyses (HR 1.03 per unit increase, 95% CI 1.01–1.05, *p* = 0.002), also after adjusting for age, coronary risk factors and comorbidity (RR 1.02, 95% CI 1.00–1.05, *p* = 0.037). After further adjustment for symptoms of anxiety and depression there was only a week, not significant association between NA and MACE (RR 1.01, 95% CI 0.98–1.05, *p* = 0.53). The analyses with imputed data revealed no considerable differences.

**Table 2:** Hazard ratio [HR] for major adverse cardiovascular events (MACE) in patients with coronary artery disease (CAD).

	Model 1^1^		Model 2^2^		Model 3^3^		Model 4^4^	
	MACE		MACE		MACE		MACE	
Outcome variables	HR (95% CI)	*p*-value	HR (95% CI)	*p*-value	HR (95% CI)	*p*-value	HR (95% CI)	*p*-value
Type D personality	1.25 (0.91–1.71)	0.171	1.20 (0.86–1.66)	0.289	1.10 (0.78–1.56)	0.571	0.88 (0.59–1.31)	0.527
Negative affectivity	1.03 (1.01–1.05)	0.002	1.03 (1.01–1.05)	0.007	1.02 (1.00–1.05)	0.037	1.01 (0.98–1.05)	0.532
Social inhibition	1.01 (0.99–1.04)	0.291	1.01 (0.99–1.04)	0.320	1.01 (0.99–1.03)	0.442	1,00 (0.97–1.02)	0.759

MACE, Major adverse cardiovascular events.

1 Adjusted for age. Analysis is stratified by prior coronary events before the index event or not.

2 Adjusted for coronary risk factors with value of p < =0.1 in crude or age adjusted analyses (smoking, LDL cholesterol, physical activity and systolic blood pressure) in addition to adjustments in Model 1.

3 Adjusted for cardiovascular comorbidity with *p*-value ≤ 0.1 in crude analyses (stroke, peripheral artery disease, and kidney failure) in addition to adjustments in Model 2.

4 Adjusted for anxiety and depression in addition to adjustments in model 3.

We observed that many of the risk factors were more prevalent in the Type D and high NA groups compared to those without Type D or with high SI only (Table 3). Furthermore, anxiety and depression scores were significantly higher in the high NA group than in the high SI group. Cronbach’s αs were 0.87 for NA, 0.86 for SI, 0.84 for HADS-A and 0.76 for HADS-D.

**Table 3:** A comparison of clinical and psychological factors in Type D subgroups.

	1. NA−/SI− (*n* = 576)	2. SI+/NA− (*n* = 183)	3. NA+/SI− (*n* = 127)	4. NA+/SI+ (*n* = 197)	*p*-value for differences between groups
**Socio-demographic factors**					
Age at index event, mean (SD)	62.4 (9.3)	63.1 (8.8)	59.1 (9.6)	59.3 (10.5)	<0.001
Female sex, % (*n*)	18.2 (105)	18.6 (34)	28.3 (36)	26.4 (52)	0.012
**Clinical factors**					
Participation in cardiac rehabilitation, % (*n*)	53.3 (307)	55.2 (101)	53.5 (68)	9.7 (98)	0.748
Not taking statins last week, % (*n*)	4.5 (26)	1.7 (3)	7.9 (10)	7.3 (14)	0.031
Smoking at inclusion, % (*n*)	18.6 (103)	16.6 (29)	24.0 (29)	29.0 (56)	0.007
Low physical activity,^1^ % (*n*)	15.7 (88)	18.1 (33)	18.1 (23)	23.2 (45)	0.128
Systolic blood pressure (mmHg), mean (SD)	139.5 (19.5)	139.5 (17.8)	135.1 (19.1)	135.5 (17.9)	0.021
LDL (mmol/L), mean (SD)	2.1 (0.7)	2.0 (0.7)	2.2 (0.9)	2.1 (0.8)	0.095
**Psychological factors**					
HADS—depression, mean (SD)	2.4 (2.2)	3.7 (2.5)	5.4 (3.4)	8.4 (3.7)	<0.001
HADS—anxiety, mean (SD)	3.1 (2.5)	3.9 (2.8)	7.6 (3.5)	7.0 (3.5)	<0.001

SD, Standard deviation; *n*, number; LDL, low-density lipoprotein; N.S, not significant; NA, negative affectivity; SI, social inhibition; HADS, hospital anxiety and depression scale.^*^p < 0.05, ^**^p < 0.01, ^***^p < 0.001.

1 Physical activity less than 1 time weekly.

## Discussion

The main finding was an association between higher NA scores and an increased risk of recurrent CVD events in outpatients with CAD. SI was not clearly associated with the risk of recurrent CVD events. Furthermore, we found associations with Type D, smoking and not taking statins. Particularly high NA/low SI and Type D were associated with low statin adherence and CVD risk factors (Supplementary Table 1). To the best of our knowledge, this is the first study that investigates the associations between CVD risk factors and high NA/low SI respective high SI/low NA subgroups.

Our findings suggest that NA may be the most important independent prognostic trait of Type D personality regarding the long-term risk or recurrent MACE in CAD patients. This is consistent with results from some studies (Denollet and Brutsaert, 1998; Wang et al., 2018), but contrary to others (Denollet and Pedersen, 2008; Meyer et al., 2014; Kupper and Denollet, 2016; Conden et al., 2017). Type D may relates to MACE through NA, with no considerable additional effect of SI. The association between NA and prognosis was no longer significant after adjustments for anxiety and depression. This may be due to the NA construct overlapping with depression and anxiety or an underlying dimension between depression, anxiety and NA. A recent study indicated that there may be such an underlying dimension, particularly between HADS-D and NA (Tunheim et al., 2022). Moreover, Type D personality may render the person vulnerable to experiencing higher levels of anxiety and depression, which may be a consequence to being diagnosed with CAD. We found a significant correlation of 0.63 between NA and depression and 0.73 between NA and anxiety. These correlations between NA and depression and NA and anxiety limits our statistical power in multivariate analysis.

One study with assessment of Type D at 6 months after PCI reported a significant association between outcome (MI or all-cause mortality) and Type D personality (Pedersen et al., 2004). However, the outcome variable differed from our study and the follow up period was significantly shorter (9 months). We may speculate that Type D affect CVD prognosis according to the duration of follow up after an acute event, although there are contradictory results whether Type D affects short or long-term prognosis. One study identified a higher risk for re-stenosis after 2 years compared to 1 year (Wang et al., 2018), but we are not aware of any other study with comparisons of prognostic differences according to various follow up durations. Type D has been identified as an independent factor associated with recurrent MI or all-cause mortality in post-acute MI patients. The association is stronger in younger (<70 years) compared to older patients (Denollet et al., 2013b), and a recent study identified Type D as a negative prognostic factors in young (<55 years) MI patients (Wang et al., 2022). We did not find a significant association between overall Type D and MACE, but this might very well be due to limited statistical power, as confidence intervals are wide and there where a statistical significant association with NA.

Others have reported significant associations between Type D personality and poor CVD prognosis in CAD patients, also when controlling for depression (Denollet et al., 1995, 1996, 2000, 2006a; Denollet and Brutsaert, 1998; Denollet and Pedersen, 2008; Martens et al., 2010; Vukovic et al., 2014; Wang et al., 2018). This result is in alignment with the review by Grande et al. reporting that the pooled effect for six studies on Type D and prognosis in CAD controlling for psychological symptoms, no longer found an independent effect of Type D (Grande et al., 2012).

We found a significant relationship between Type D and higher prevalence of smoking. This is in line with other studies showing that smoking behavior has been associated with Type D personality in patients with (Svansdottir et al., 2012) and without CAD (Einvik et al., 2011; Gilmour and Williams, 2012), whereas other studies have failed to find such an association (Williams et al., 2008; Mommersteeg et al., 2010). Regarding the association of Type D and smoking, our results are in line with those found in apparently healthy individuals (Gilmour and Williams, 2012; Wiencierz and Williams, 2017). Altogether, smoking and statin non-adherence were also found to be among the strongest predictors of MACE in a previous study from our group (Sverre et al., 2020). Potential interventions to improve outcomes in these patients should therefore probably aim at modifying smoking and statin non-adherence. Interestingly, a recent lifestyle intervention study also showed an impact on Type D personality (Kim et al., 2021).

Low adherence to CVD medication is strongly associated with poor prognosis in CAD patients (Visseren et al., 2021). In line with previous studies, we found that Type D was associated with low adherence to statins (Wu et al., 2013, 2015; Crawshaw et al., 2016; Wang et al., 2018). Adherence was most strongly associated with NA. Our results are in agreement with other studies report that NA explains 23% of the variation in medication non-adherence 3 months after MI (Gilmour and Williams, 2012) and is the only Type D trait significantly associated with medication non-adherence 6 months after hospitalization for MI (Molloy et al., 2012). Thus, in studies investigating NA separately, the results consistently indicate that NA is the Type D trait that drives the association between Type D and non-adherence. Moreover, poor adherence to aspirin and statins was reported as an independent predictor of in-stent restenosis and potentially mediated the association between Type D and in-stent restenosis (Wang et al., 2018). Consequently, the role of Type D and particularly NA and their associations to adherence and in-stent restenosis should be addressed in future studies.

It has been hypothesized that the relationship between Type D personality and medication non-adherence might be explained by inadequate consultation behavior, possibly due to the SI component associated with fear of disapproval and rejection by others (Gilmour and Williams, 2012). In turn, this may impact the patient-doctor relationship though impaired communication (Gilmour and Williams, 2012). However, our study delineating the specific contributions of NA and SI to risk factors, indicate that SI might not be the driver of non-adherence. This suggests that the effect of NA on prognosis partly may be mediated through higher prevalence of CVD risk factors.

In total, our results indicate that NA measured by DS14 may represent a personality variable that may aid in identifying CAD patients at high risk of poor self-management. Personality traits such as NA are considered to exert a stable influence on behavior, and the level of distress or NA of patients with Type D personality can be modified. Recently, a psychological intervention targeting personality traits such as neuroticisms rather than psychological disorders has been developed showing effects on the score of NA (Sauer-Zavala et al., 2012; Barlow et al., 2017; Sauer-Zavala et al., 2017). This is interesting because Type D personality has been highly correlated with neuroticism (De Fruyt and Denollet, 2002). Together with our results, suggesting a potential overlap between depression, anxiety and NA, further studies on the treatment for depression and anxiety in patients with CAD may also assess the effectiveness on NA and SI as well as Type D. Recently, the attention training technique—a component of metacognitive therapy—showed effect on anxiety, depression and NA in these patients (Dammen et al., 2022). Whether the attention training technique also influences the presence of Type D as well as the risk for recurrent adverse events in CAD patients remains to be investigated. Future intervention studies should also include assessments of suggested psychological (cognitive appraisal and coping style; Lv et al., 2020) as well as pathophysiological mechanisms responsible for the adverse effect of NA or Type D.

## Strengths and limitations

Strengths of the study include a high participation rate and a representative sample of patients with chronic CAD from routine clinical practice. MACE were extracted from hospital medical records by experienced cardiologists and only 14 patients (1%) were lost to follow-up. Limitations are that some registration of MACE may have been missed occurring outside the catchment area of the participating hospitals. However, the local hospital record system usually gets a report on such events. The hospital medical record is coupled to the Population Registry in Norway with weekly updates, and it is unlikely that any deaths have been missed. Because patients were included 2–36 months after the index event, 160 patients had died between the time of event and inclusion. Hence, our results might not be valid for the first months following an index event. Even though we have evaluated a broad spectrum of possible determinants associated with MACE, additional confounders such as anger and insomnia may be considered in future studies. Some confounding and multi-collinearity in the multivariate analyses must be assumed as NA is very closely associated with anxiety and depression (Iqbal and Dar, 2015). Overlapping measurements and constructs make it difficult to determine whether different personality is associated with CAD risk. More research is needed to clarify to what degree these constructs overlap. In addition, statin non-adherence was assessed by self-report only and we did not have objective methods for this assessment.

## Conclusion

Our results indicate that negative affectivity is associated with recurrent major adverse cardiovascular events both before and after adjustment for comorbidity and cardiovascular risk factors, while we found little relationship to social inhibition. Negative affectivity was also associated with some of the most unfavorable lifestyle factors for CAD prognosis (smoking, statin non-adherence) and depression. Consequently, negative affectivity may be a marker of poor lifestyle and psychological distress and thus a potential important factor to screen for in identifying high-risk CAD outpatients in need of individualized treatment.

## Data availability statement

The datasets presented in this article are not readily available because according to Norwegian legislation, the Norwegian Data Protection Authority, and the Committee of Ethics, we are not allowed to share original study data publicly. However, the essential generated data are available from the corresponding author on reasonable request. Requests to access the datasets should be directed to k.s.torgersen@medisin.uio.no.

## Ethics statement

The studies involving human participants were reviewed and approved by the Regional Committee of Ethics for Medical and Health Research Gullhaug Torg 4 Oslo, 0484. The patients/participants provided their written informed consent to participate in this study.

## Author contributions

TD and JM contributed to the study design. KT, HW-F, and ES carried out the analyses. KT, ES, HW-F, OA, JM, and TD contributed to the interpretation of the data. KT drafted the manuscript. All authors contributed to the article and approved the submitted version.

## Funding

KT received founding from the University of Oslo reference number 2018/4606.

## Conflict of interest

The authors declare that the research was conducted in the absence of any commercial or financial relationships that could be construed as a potential conflict of interest.

## Publisher’s note

All claims expressed in this article are solely those of the authors and do not necessarily represent those of their affiliated organizations, or those of the publisher, the editors and the reviewers. Any product that may be evaluated in this article, or claim that may be made by its manufacturer, is not guaranteed or endorsed by the publisher.

## References

[ref1] Al-Qezweny M. N. A. Utens E. M. W. J. Dulfer K. Hazemeijer B. A. F. van Geuns R. J. Daemen J.. (2016). The association between type D personality, and depression and anxiety ten years after PCI. Netherlands Heart J. 24, 538–543. doi: 10.1007/s12471-016-0860-4, PMID: 27294841 PMC5005192

[ref2] Barlow D. H. Farchione T. J. Bullis J. R. Gallagher M. W. Murray-Latin H. Sauer-Zavala S.. (2017). The unified protocol for Transdiagnostic treatment of emotional disorders compared with diagnosis-specific protocols for anxiety disorders: a randomized clinical trial. JAMA Psychiat. 74, 875–884. doi: 10.1001/jamapsychiatry.2017.2164, PMID: 28768327 PMC5710228

[ref3] Bergvik S. Sorlie T. Wynn R. Sexton H. (2010). Psychometric properties of the type D scale (DS14) in Norwegian cardiac patients. Scand. J. Psychol. 51, 334–340. doi: 10.1111/j.1467-9450.2009.00793.x, PMID: 20102545

[ref4] Bjelland I. Dahl A. A. Haug T. T. Neckelmann D. (2002). The validity of the hospital anxiety and depression scale. J. Psychosom. Res. 52, 69–77. doi: 10.1016/s0022-3999(01)00296-3 11832252

[ref5] Carney R. M. Freedland K. E. (2017). Depression and coronary heart disease. Nat. Rev. Cardiol. 14, 145–155. doi: 10.1038/nrcardio.2016.181 27853162

[ref6] Christodoulou C. Douzenis A. Mommersteeg P. M. Rallidis L. Poulios A. Efstathiou V.. (2013). A case-control validation of type D personality in Greek patients with stable coronary heart disease. Ann. Gen. Psychiatry 12:38. doi: 10.1186/1744-859x-12-38, PMID: 24283252 PMC4175478

[ref7] Conden E. Rosenblad A. Wagner P. Leppert J. Ekselius L. Aslund C. (2017). Is type D personality an independent risk factor for recurrent myocardial infarction or all-cause mortality in post-acute myocardial infarction patients? Eur. J. Prev. Cardiol. 24, 522–533. doi: 10.1177/2047487316687427, PMID: 28071958

[ref8] Crawshaw J. Auyeung V. Norton S. Weinman J. (2016). Identifying psychosocial predictors of medication non-adherence following acute coronary syndrome: a systematic review and meta-analysis. J. Psychosom. Res. 90, 10–32. doi: 10.1016/j.jpsychores.2016.09.003, PMID: 27772555

[ref9] Dammen T. Tunheim K. Munkhaugen J. Papageorgiou C. (2022). The attention training technique reduces anxiety and depression in patients with coronary heart disease: a pilot feasibility study. Front. Psychol. 13:948081. doi: 10.3389/fpsyg.2022.948081, PMID: 35967654 PMC9363691

[ref10] De Fruyt F. Denollet J. (2002). Type D personality: a five-factor model perspective. Psychol. Health 17, 671–683. doi: 10.1080/08870440290025858

[ref11] Denollet J. (2005). DS14: standard assessment of negative affectivity, social inhibition, and type D personality. Psychosom. Med. 67, 89–97. doi: 10.1097/01.psy.0000149256.81953.49, PMID: 15673629

[ref12] Denollet J. Brutsaert D. L. (1998). Personality, disease severity, and the risk of long-term cardiac events in patients with a decreased ejection fraction after myocardial infarction. Circulation 97, 167–173. doi: 10.1161/01.cir.97.2.167, PMID: 9445169

[ref13] Denollet J. Conraads V. M. (2011). Type D personality and vulnerability to adverse outcomes in heart disease. Cleve. Clin. J. Med. 78, S13–S19. doi: 10.3949/ccjm.78.s1.02, PMID: 21972324

[ref14] Denollet J. Pedersen S. S. (2008). Prognostic value of type D personality compared with depressive symptoms. Arch. Intern. Med. 168, 431–432. doi: 10.1001/archinternmed.2007.120, PMID: 18299500

[ref15] Denollet J. Pedersen S. S. Ong A. T. Erdman R. A. Serruys P. W. van Domburg R. T. (2006a). Social inhibition modulates the effect of negative emotions on cardiac prognosis following percutaneous coronary intervention in the drug-eluting stent era. Eur. Heart J. 27, 171–177. doi: 10.1093/eurheartj/ehi616, PMID: 16246826

[ref16] Denollet J. Pedersen S. S. Vrints C. J. Conraads V. M. (2006b). Usefulness of type D personality in predicting five-year cardiac events above and beyond concurrent symptoms of stress in patients with coronary heart disease. Am. J. Cardiol. 97, 970–973. doi: 10.1016/j.amjcard.2005.10.035, PMID: 16563897

[ref17] Denollet J. Pedersen S. S. Vrints C. J. Conraads V. M. (2013a). Predictive value of social inhibition and negative affectivity for cardiovascular events and mortality in patients with coronary artery disease: the type D personality construct. Psychosom. Med. 75, 873–881. doi: 10.1097/psy.0000000000000001, PMID: 24163388

[ref18] Denollet J. Sys S. U. Brutsaert D. L. (1995). Personality and mortality after myocardial infarction. Psychosom. Med. 57, 582–591. doi: 10.1097/00006842-199511000-00011 8600485

[ref19] Denollet J. Sys S. U. Stroobant N. Rombouts H. Gillebert T. C. Brutsaert D. L. (1996). Personality as independent predictor of long-term mortality in patients with coronary heart disease. Lancet 347, 417–421. doi: 10.1016/s0140-6736(96)90007-0 8618481

[ref20] Denollet J. Tekle F. B. van der Voort P. H. Alings M. van den Broek K. C. (2013b). Age-related differences in the effect of psychological distress on mortality: type D personality in younger versus older patients with cardiac arrhythmias. Biomed. Res. Int. 2013:246035. doi: 10.1155/2013/246035, PMID: 24205502 PMC3800613

[ref21] Denollet J. Vaes J. Brutsaert D. L. (2000). Inadequate response to treatment in coronary heart disease: adverse effects of type D personality and younger age on 5-year prognosis and quality of life. Circulation 102, 630–635. doi: 10.1161/01.cir.102.6.630 10931802

[ref22] Donders A. R. van der Heijden G. J. Stijnen T. Moons K. G. (2006). Review: a gentle introduction to imputation of missing values. J. Clin. Epidemiol. 59, 1087–1091. doi: 10.1016/j.jclinepi.2006.01.014, PMID: 16980149

[ref23] Du J. Zhang D. Yin Y. Zhang X. Li J. Liu D.. (2016). The personality and psychological stress predict major adverse cardiovascular events in patients with coronary heart disease after percutaneous coronary intervention for five years. Medicine 95:e3364. doi: 10.1097/md.0000000000003364, PMID: 27082597 PMC4839841

[ref24] Dulfer K. Hazemeijer B. A. van Dijk M. R. van Geuns R. J. M. Daemen J. van Domburg R. T.. (2015). Prognostic value of type D personality for 10-year mortality and subjective health status in patients treated with percutaneous coronary intervention. J. Psychosom. Res. 79, 214–221. doi: 10.1016/j.jpsychores.2015.05.014, PMID: 26084732

[ref25] Einvik G. Dammen T. Hrubos-Strøm H. Namtvedt S. K. Randby A. Kristiansen H. A.. (2011). Prevalence of cardiovascular risk factors and concentration of C-reactive protein in type D personality persons without cardiovascular disease. Eur. J. Cardiovasc. Prev. Rehabil. 18, 504–509. doi: 10.1177/1741826710389383, PMID: 21450648

[ref26] Garcia-Retamero R. Petrova D. Arrebola-Moreno A. Catena A. Ramírez-Hernández J. A. (2016). Type D personality is related to severity of acute coronary syndrome in patients with recurrent cardiovascular disease. Br. J. Health Psychol. 21, 694–711. doi: 10.1111/bjhp.12196, PMID: 27222488

[ref27] Gilmour J. Williams L. (2012). Type D personality is associated with maladaptive health-related behaviours. J. Health Psychol. 17, 471–478. doi: 10.1177/1359105311423117, PMID: 21975661

[ref28] Grande G. Romppel M. Barth J. (2012). Association between type D personality and prognosis in patients with cardiovascular diseases: a systematic review and meta-analysis. Ann. Behav. Med. 43, 299–310. doi: 10.1007/s12160-011-9339-0, PMID: 22237826

[ref29] Grande G. Romppel M. Vesper J. M. Schubmann R. Glaesmer H. Herrmann-Lingen C. (2011). Type D personality and all-cause mortality in cardiac patients--data from a German cohort study. Psychosom. Med. 73, 548–556. doi: 10.1097/PSY.0b013e318227a9bc 21862827

[ref30] Imbalzano E. Vatrano M. Quartuccio S. Ceravolo R. Ciconte V. A. Rotella P.. (2018). Effect of type D personality on smoking status and their combined impact on outcome after acute myocardial infarction. Clin. Cardiol. 41, 321–325. doi: 10.1002/clc.22865, PMID: 29457844 PMC6489971

[ref31] Iqbal N. Dar K. A. (2015). Negative affectivity, depression, and anxiety: does rumination mediate the links? J. Affect. Disord. 181, 18–23. doi: 10.1016/j.jad.2015.04.002, PMID: 25913918

[ref32] Kim E. J. Nho J. H. Kim H. Y. Park S. K. (2021). The effects of lifestyle interventions on the health-promoting behavior, type D personality, cognitive function and body composition of low-income middle-aged Korean women. Int. J. Environ. Res. Public Health 18. doi: 10.3390/ijerph18115637, PMID: 34070377 PMC8197549

[ref33] Kupper N. Denollet J. (2014). Type D personality is associated with social anxiety in the general population. Int. J. Behav. Med. 21, 496–505. doi: 10.1007/s12529-013-9350-x, PMID: 24072352

[ref34] Kupper N. Denollet J. (2016). Explaining heterogeneity in the predictive value of type D personality for cardiac events and mortality. Int. J. Cardiol. 224, 119–124. doi: 10.1016/j.ijcard.2016.09.006, PMID: 27648980

[ref35] Kupper N. Denollet J. (2018). Type D personality as a risk factor in coronary heart disease: a review of current evidence. Curr. Cardiol. Rep. 20:104. doi: 10.1007/s11886-018-1048-x, PMID: 30209683 PMC6153564

[ref36] Leiknes K. A. Siqveland J. (2016). Folkehelseinstituttets gjennomgang av HADS. Oslo, Norway: Folkehelseinstituttet.

[ref37] Lloyd-Jones D. Adams R. J. Brown T. M. Carnethon M. Dai S. De Simone G.. (2010). Executive summary: heart disease and stroke statistics--2010 update: a report from the American Heart Association. Circulation 121, 948–954. doi: 10.1161/circulationaha.109.192666 20177011

[ref38] Lv H. Tao H. Wang Y. Zhao Z. Liu G. Li L.. (2020). Impact of type D personality on major adverse cardiac events in patients undergoing percutaneous coronary intervention: the mediating role of cognitive appraisal and coping style. J. Psychosom. Res. 136:110192:110192. doi: 10.1016/j.jpsychores.2020.110192, PMID: 32721776

[ref39] Majaluoma S. Seppälä T. Kautiainen H. Korhonen P. (2020). Type D personality and metabolic syndrome among Finnish female municipal workers. BMC Womens Health 20:202. doi: 10.1186/s12905-020-01052-z, PMID: 32928173 PMC7489202

[ref40] Marchesi C. Ossola P. Scagnelli F. Paglia F. Aprile S. Monici A.. (2014). Type D personality in never depressed patients at their first acute coronary syndrome. Psychother. Psychosom. 83, 190–191. doi: 10.1159/000358525, PMID: 24752175

[ref41] Martens E. J. Mols F. Burg M. M. Denollet J. (2010). Type D personality predicts clinical events after myocardial infarction, above and beyond disease severity and depression. J. Clin. Psychiatry 71, 778–783. doi: 10.4088/JCP.08m04765blu, PMID: 20156412

[ref42] Meyer T. Hussein S. Lange H. W. Herrmann-Lingen C. (2014). Type D personality is unrelated to major adverse cardiovascular events in patients with coronary artery disease treated by intracoronary stenting. Ann. Behav. Med. 48, 156–162. doi: 10.1007/s12160-014-9590-2, PMID: 24481867

[ref43] Molloy G. J. Randall G. Wikman A. Perkins-Porras L. Messerli-Burgy N. Steptoe A. (2012). Type D personality, self-efficacy, and medication adherence following an acute coronary syndrome. Psychosom. Med. 74, 100–106. doi: 10.1097/PSY.0b013e31823a5b2f, PMID: 22155940

[ref44] Mommersteeg P. M. Kupper N. Denollet J. (2010). Type D personality is associated with increased metabolic syndrome prevalence and an unhealthy lifestyle in a cross-sectional Dutch community sample. BMC Public Health 10:714. doi: 10.1186/1471-2458-10-714, PMID: 21092104 PMC3002331

[ref45] Munkhaugen J. Sverre E. Peersen K. Gjertsen E. Gullestad L. Moum T.. (2016). The role of medical and psychosocial factors for unfavourable coronary risk factor control. Scand. Cardiovasc. J. 50, 1–8. doi: 10.3109/14017431.2015.1111408, PMID: 26488672

[ref46] Pedersen S. S. Denollet J. Ong A. T. Sonnenschein K. Erdman R. A. Serruys P. W.. (2007). Adverse clinical events in patients treated with sirolimus-eluting stents: the impact of type D personality. Eur. J. Cardiovasc. Prev. Rehabil. 14, 135–140. doi: 10.1097/HJR.0b013e328045c282, PMID: 17301639

[ref47] Pedersen S. S. Lemos P. A. van Vooren P. R. Liu T. K. Daemen J. Erdman R. A.. (2004). Type D personality predicts death or myocardial infarction after bare metal stent or sirolimus-eluting stent implantation: a rapamycin-eluting stent evaluated at Rotterdam cardiology hospital (RESEARCH) registry substudy. J. Am. Coll. Cardiol. 44, 997–1001. doi: 10.1016/j.jacc.2004.05.064 15337209

[ref48] Peersen K. Munkhaugen J. Gullestad L. Dammen T. Moum T. Otterstad J. E. (2017). Reproducibility of an extensive self-report questionnaire used in secondary coronary prevention. Scand. J. Public Health 45, 269–276. doi: 10.1177/1403494816688375, PMID: 28181463 PMC5405837

[ref49] Raykh O. I. Sumin A. N. Korok E. V. (2021). The influence of personality type D on cardiovascular prognosis in patients after coronary artery bypass grafting: data from a 5-year-follow-up study. Int. J. Behav. Med. 29, 46–56. doi: 10.1007/s12529-021-09992-y, PMID: 33954890 PMC8099536

[ref50] Sauer-Zavala S. Boswell J. F. Gallagher M. W. Bentley K. H. Ametaj A. Barlow D. H. (2012). The role of negative affectivity and negative reactivity to emotions in predicting outcomes in the unified protocol for the transdiagnostic treatment of emotional disorders. Behav. Res. Ther. 50, 551–557. doi: 10.1016/j.brat.2012.05.005, PMID: 22738907 PMC3408841

[ref51] Sauer-Zavala S. Wilner J. G. Barlow D. H. (2017). Addressing neuroticism in psychological treatment. Personal Disord 8, 191–198. doi: 10.1037/per0000224 29120218

[ref52] Svansdottir E. van den Broek K. C. Karlsson H. D. Gudnason T. Denollet J. (2012). Type D personality is associated with impaired psychological status and unhealthy lifestyle in Icelandic cardiac patients: a cross-sectional study. BMC Public Health 12:42. doi: 10.1186/1471-2458-12-42, PMID: 22251667 PMC3398279

[ref53] Sverre E. Peersen K. Weedon-Fekjær H. Perk J. Gjertsen E. Husebye E.. (2020). Preventable clinical and psychosocial factors predicted two out of three recurrent cardiovascular events in a coronary population. BMC Cardiovasc. Disord. 20:61. doi: 10.1186/s12872-020-01368-6, PMID: 32024471 PMC7003324

[ref54] Tunheim K. Dammen T. Baardstu S. Moum T. Munkhaugen J. Papageorgiou C. (2022). Relationships between depression, anxiety, type D personality, and worry and rumination in patients with coronary heart disease. Front. Psychol. 13:929410. doi: 10.3389/fpsyg.2022.929410, PMID: 36186321 PMC9517376

[ref55] Visseren F. L. J. Mach F. Smulders Y. M. Carballo D. Koskinas K. C. Bäck M.. (2021). 2021 ESC guidelines on cardiovascular disease prevention in clinical practice: developed by the task force for cardiovascular disease prevention in clinical practice with representatives of the European Society of Cardiology and 12 medical societies with the special contribution of the European Association of Preventive Cardiology (EAPC). Eur. Heart J. 42, 3227–3337. doi: 10.1093/eurheartj/ehab484, PMID: 34458905

[ref56] Vukovic O. Tosevski D. L. Jasovic-Gasic M. Damjanovic A. Zebic M. Britvic D.. (2014). Type D personality in patients with coronary artery disease. Psychiatr. Danub. 26, 46–51. PMID: 24608156

[ref57] Wang Y. Gao X. Zhao Z. Li L. Liu G. Yu B.. (2022). Predictive value of type D personality for cardiovascular events in young patients with acute myocardial infarction: a prospective, observational study. Eur. J. Prev. Cardiol. 29, e100–e101. doi: 10.1093/eurjpc/zwab030, PMID: 34333587

[ref58] Wang Y. Liu G. Gao X. Zhao Z. Li L. Chen W.. (2018). Prognostic value of type D personality for in-stent restenosis in coronary artery disease patients treated with drug-eluting stent. Psychosom. Med. 80, 95–102. doi: 10.1097/psy.0000000000000532, PMID: 28938244

[ref59] Wiencierz S. Williams L. (2017). Type D personality and physical inactivity: the mediating effects of low self-efficacy. J. Health Psychol. 22, 1025–1034. doi: 10.1177/1359105315622557, PMID: 26837688

[ref60] Williams L. O'Connor R. C. Howard S. Hughes B. M. Johnston D. W. Hay J. L.. (2008). Type-D personality mechanisms of effect: the role of health-related behavior and social support. J. Psychosom. Res. 64, 63–69. doi: 10.1016/j.jpsychores.2007.06.008, PMID: 18158001

[ref61] Wu J. R. Lennie T. A. Dekker R. L. Biddle M. J. Moser D. K. (2013). Medication adherence, depressive symptoms, and cardiac event-free survival in patients with heart failure. J. Card. Fail. 19, 317–324. doi: 10.1016/j.cardfail.2013.03.010, PMID: 23663814 PMC3699198

[ref62] Wu J. R. Song E. K. Moser D. K. (2015). Type D personality, self-efficacy, and medication adherence in patients with heart failure-a mediation analysis. Heart Lung 44, 276–281. doi: 10.1016/j.hrtlng.2015.03.006, PMID: 25979573 PMC4470745

[ref63] Zigmond A. S. Snaith R. P. (1983). The hospital anxiety and depression scale. Acta Psychiatr. Scand. 67, 361–370. doi: 10.1111/j.1600-0447.1983.tb09716.x 6880820

