# Supplementary material for: Risk of recurrent cardiovascular events in coronary artery disease patients with Type D personality
Source: Front Psychol. 2023 Mar 28;14:1119146. doi: 10.3389/fpsyg.2023.1119146 (PMC10088369; doi:10.3389/fpsyg.2023.1119146)
Supplement: Supplementary file 1 [file Table_1.docx]

**Supplementary Table 1. Baseline characteristics of DS14-responders vs non-responders**

|  | **Non-responders (n=44) % (n)** | **Responders (n=1083) % (n)** | **p-value** |  |  |
| --- | --- | --- | --- | --- | --- |
|  |  |  |  |  |  |
| ***Socio-demographic factors*** |  |  |  |  |  |
| Age at index event, mean (SD) | 63.9 (1.0) | 63.6 (9.6) | 0.110 |  |  |
| Female sex, % (n) | 23.3 (10) | 21.0 (227) | 0.715 |  |  |
| Living alone, % (n) | 18.8 (8) | 19.2 (194) | 0.935 |  |  |
| Low education ^1^, % (n) | 72.0 (27) | 70.1 (752) | 0.714 |  |  |
| ***Medical factors*** |  |  |  |  |  |
| Myocardial infarction, % (n) | 83.7 (36) | 79.3 (860) | 0.485 |  |  |
| Stable or unstable angina, % (n) | 16.3 (7) | 20.6 (223) | 0.485 |  |  |
| More than 1 previous coronary event, % (n) | 14.0 (6) | 14.4 (156) | 0.936 |  |  |
| Heart failure, % (n) | 18.6 (8) | 12.9 (140) | 0.279 |  |  |
| Peripheral artery disease, % (n) | 0 | 8.6 (93) | 0.082 |  |  |
| Stroke or transient ischemic attack, % (n) | 21.4 (3) | 6.9 (75) | 0.238 |  |  |
| Chronic kidney failure (eGFR <60 mL/min/1.73m 2), % (n) | 0 | 13.3 (132) | 0.530 |  |  |
| Participation in cardiac rehabilitation, % (n) | 60.5 (26) | 53.0 (574) | 0.339 |  |  |
| Not using statins at inclusion, % (n) | 14.3 (2) | 7.2 (78) | 0.149 |  |  |
| Current smoking^2^, % (n) | 33.3 (13) | 20.8 (217) | 0.060 |  |  |
| Low physical activity^3^ , % (n) | 73.5 (25) | 59.5 (640) | 0.100 |  |  |
| Systolic blood pressure (mmHg), mean (SD) | 138 (20.8) | 138 (19.0) | 0.991 |  |  |
| LDL-cholesterol (mmol/L), mean (SD) | 2.2 (0.9) | 2.1 (0.8) | 0.378 |  |  |
| Diabetes, % (n) | 0 | 16.6 (180) | 0.077 |  |  |
| C-reactive protein, mean (SD) | 3.1(2.6) | 2.5 (2.9) | 0.091 |  |  |
| Central obesity^4^, % (n) | 71.4 (25) | 59.4 (579) | 0.151 |  |  |
| ***Psychological factors*** |  |  |  |  |  |
| DS14 negative affection (score 0–28), mean (SD) | - | 7.0 (5.9) | - |  |  |
| DS14 social inhibition (score 0–28), mean (SD) | - | 7.5 (5.7) | - |  |  |
| HADS - depression, mean (SD) | 5.4 (4.3) | 3.8 (3.2) | 0.076 |  |  |
| HADS - anxiety, mean (SD) | 6.7 (4.6) | 4.7 (3.7) | 0.013 |  |  |
| SD: standard deviation; n: number, LDL: low density lipoprotein, eGFR: estimated glomerular filtration rate. HADS: hospital anxiety and depression scale. |  |  |  |  |  |
| **^1^**Completion of primary or secondary school only |  |  |  |  |  |
| **^2^**Smoking at inclusion |  |  |  |  |  |
| **^3^**Physical activity less than 30 minutes of moderate activity 2-3 times weekly |  |  |  |  |  |
| **^4^**Waist circumference ≥102cm in males and ≥88 cm in females | | | |  |  |
|  | |  |  |  |  |
